# Differential effect of wealth quintile on modern contraceptive use and fertility: evidence from Malawian women

**DOI:** 10.1186/1472-6874-14-40

**Published:** 2014-03-07

**Authors:** Stephen A Adebowale, Sunday A Adedini, Latifat D Ibisomi, Martin E Palamuleni

**Affiliations:** 1 Population Training and Research Unit, Faculty of Humanities and Social Sciences, North-West University, Mafikeng, South Africa; 2 Department of Epidemiology and Medical Statistics, Faculty of Public Health, College of Medicine, University of Ibadan, Ibadan, Nigeria; 3 Department of Demography and Social Statistics, Faculty of Social Sciences, Obafemi Awolowo University, Ile-Ife, Nigeria; 4 Demography and Population Studies Programme, School of Social Sciences, Faculty of Humanities, University of the Witwatersrand, Johannesburg, South Africa

**Keywords:** Modern contraceptive use, Richest and lowest wealth quintile, Total fertility rate

## Abstract

**Background:**

High fertility and wide inequality in wealth distribution are phenomenal problems in sub-Saharan Africa. Modern Contraceptives (MC) are useful for limiting fertility, but are not always easily accessible in Malawi. This study examines the gap in MC use and fertility between women in the richest and poorest Wealth Quintile (WQ).

**Methods:**

The study was cross-sectional in design and utilized Malawi DHS dataset, 2010. It focused on women of reproductive age. The dependent variables are ever and current use of MC. Chi-square and multinomial logistic regression were used for the analysis.

**Results:**

Mean children ever born by women in the poorest and richest WQs were 3.94 ± 2.7 and 2.82 ± 2.3 respectively (p < 0.001). The adjusted total fertility rate (Adj.TFR) was higher among women in the poorest (Adj.TFR = 7.60) WQ than the richest (Adj.TFR = 4.45). The prevalence of ever use of MC was higher among women in the richest WQ (82.4%) than the poorest (66.8%) (p < 0.001). Similar pattern exists for current use of MC; 58.5% and 45.9% for women in the richest and poorest WQs respectively (p < 0.001). Women in the richest WQ were more likely to ever use (OR = 2.36; C.I = 2.07-2.69, p < 0.001) and currently using (OR = 1.66; C.I = 1.40-1.97, p < 0.001) MC than their counterparts in the poorest WQ. Slight reduction in odd-ratio of MC use among women in richest WQ resulted when socio-demographic variables were used as control.

**Conclusion:**

Fertility was higher and the use of MC was lower among women in the poorest than their counterparts in the richest WQ. Ensuring availability of MC at little or no cost may bridge the gap in contraceptive use between women in the poorest and richest WQ in Malawi.

## Background

Family planning is the conscious effort by a couple to limit or space the number of children they want to have through the use of contraceptives [1]. It improves health, reduces poverty, and empowers women [1]. Contraceptive is the main issue galvanizing unprecedented efforts to the attainment of the themes of Millennium Development Goals by year 2015 [2] and action plan of 1994 International Conference on Population and Development in Cairo [3]. However, more than 200 million women in the developing world still have unmet needs for modern contraception [4]. Such women face numerous challenges, including lack of access to information and health care services, opposition from their husbands and communities, misperceptions about side effects, and cost [5,6]. Overcoming these obstacles would increase the demand for family planning and also, about 54 million unintended pregnancies, more than 79,000 maternal deaths, and more than a million infant deaths could also be averted each year [4]. The corollary of this would mean that families may perhaps save more and begin to break the grip of poverty. This will also enable the government to make better investments in education, health care, and infrastructural development with the view to improving population health [4].

**Figure 1:**
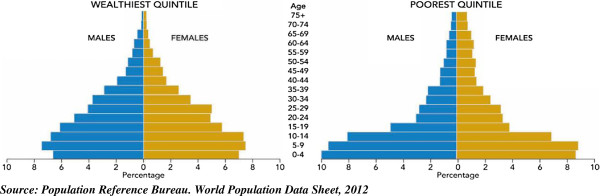
Population Pyramid of people in wealthiest and poorest wealth quintile in Malawi, 2012.

Contraceptive methods can either be modern or traditional. Family planning programmers often recommend modern contraceptive methods due to high failure rate and un-scientific nature of traditional methods [3]. Modern contraceptives have been shown to be active fertility control measure and for protection against sexually transmitted infections including HIV/AIDS [7]. In sub-Saharan Africa, aside cultural influences, utilization of modern contraceptives has been adversely affected by lack of financial capacity of individuals to acquire them when in need [8]. Modern contraceptives are often not easily accessible in most developing countries where high proportion of the people earns below one dollar per day, thus giving an edge to those who are better off financially [9,10]. Modern contraceptive use has the impetus to shape the age structure of a population. In Malawi for instance, the age structure shows that while the population of the richest is aging, that of the poor remains young [11]. One implication of this is that life-expectancy of the people in richest wealth quintile in Malawi has improved whereas that of the poor is abating (see Figure 1) [11].

In any society where there is a wide gap between socioeconomic status of the poor and the rich, the poor’s health status is compromised. Malawi has a youthful population, but its poor are much younger and its wealthy is older. In Malawi, approximately 49 percent of the household population is under age 15. However, when the age structure of Malawi is examined by wealth quintile, the richest and the poorest have quite different age structures [11-13]. Among the households in the richest quintile, about 43 percent of the population is under age 15 as against 53 percent of the poor reflecting higher level of fertility among the poorest than the richest [11,12]. The striking disparity in age composition of the population in the richest and poorest wealth quintiles calls for concern in Malawi. One begins to wonder why such variation occurs between people from different socioeconomic classes living in the same country. Many reasons or factors including contraceptive use could be responsible for the differential. This necessitates our interest to examine the level of modern contraceptive use and differential in fertility among these two groups of women in Malawi.

The focus of this paper is on women who are sexually active because these women are at risk of exposure to pregnancy and also there must be need to control their fertility. The objectives are; to examine association between socio-demographic variables and modern contraceptive use (ever and current) with particular focus on wealth quintile (poorest and richest) as key independent variable and also to know if wealth index is a predictor of modern contraceptive use (ever and current use). The total fertility rates for the women in the poorest and richest wealth quintiles were also estimated using indirect approach. Few studies in Malawi that have explored contemporary issues on contraceptive use have not adequately addressed the gap in modern contraceptive use between women in the richest and poorest wealth quintile as evidenced in our study.

## Methods

### Data collection

Malawi Demographic Health Survey (NDHS), 2010 dataset was used. The data were originally collected by Macro International in United States of America and National Statistical office Malawi. Data for this study were extracted from Measure DHS database [14]. The data is secondary; therefore detailed information about data collection procedures is contained in the MDHS 2010 report which is available on the web platform of the data originator [14]. However, a brief description of the procedures is highlighted below.

The sample for the 2010 MDHS was designed to provide population and health indicator estimates at the national, regional, and district levels. The sampling frame used for the 2010 MDHS was the 2008 Malawi Population and Housing Census (PHC), which was provided by the National Statistical Office. Administratively, Malawi is divided into 28 districts. Each district is subdivided into smaller administrative units. During the 2008 PHC, which was designed and carried out by the National Statistical Office, each of the districts was subdivided into enumeration areas (EAs), also referred to as clusters, where each EA as a whole was classified as urban or rural. The 2010 MDHS sample was selected using a stratified, two-stage cluster design, with EAs being the sampling units for the first stage. The 2010 MDHS sample included 849 clusters and a complete listing of households was done in each of the MDHS clusters. The list of households served as a sampling frame for selection of households which was the second stage of sampling. A minimum sample size of 950 households was required per district to provide an acceptable level of precision for the indicators measured in the survey and a representative sample of 27,345 households was selected for the survey [12].

### Exclusion and inclusion criteria

In this study, we analysed data on 5085 and 2290 women aged 15–49 who met the inclusion criteria set for ever and current use of contraceptive respectively. For the analysis involving ever use of contraceptive, only women who belong to either poorest or richest wealth quintile were included in the analysis while others were excluded. All women that do not provide information on contraceptive utilization status were regarded as missing values and those who have never had sexual intercourse were removed from the analysis. For current use of contraception, aside the above excluded set of women, those who are menopausal, currently pregnant, breast feeding in the last six months, not sexually active in the last four weeks before the survey were also excluded.

The dependent variables of interest were ever use and current use of modern contraceptive, whereas the independent variable of focus is wealth quintile (poorest and richest quintile). Other independent variables included; age, region, religion, education, place of residence, total life time number of sexual partners, fertility intention were used as control. All statistical tests were performed at 5% level of significant. The total fertility rates of women belonging to richest and poorest wealth quintile were also estimated to compare the difference in fertility among women in the two groups.

### Definition of key variables

Contraceptive methods are classified as modern or traditional methods. Modern methods included; female sterilisation, male sterilisation, the pill, intra-uterine device (IUD), injectables, implants, male condom, female condom, diaphragm, foam/jelly, lactational amenorrhoea method (LAM), and emergency contraception. Methods such as rhythm (periodic abstinence) and withdrawal are grouped as traditional methods. Provision was also made in the questionnaire to record any other methods mentioned by the respondent, including folk methods. Our study focused on the use of modern contraceptive methods because of their effectiveness in fertility control.

The dependent variables are:

(i) *Ever use of contraceptive method:*

This is defined as the use of contraceptive method by women at any time during or after first sexual intercourse. In this study, ever use of contraceptive method was classified as (never use = 0, traditional/folk methods = 1, modern methods = 2).

(ii) *Current use of contraceptive method:*

This is defined as the use of contraceptive method by women who had sexual intercourse in thepast four weeks prior the survey. The level of current use is a measure of actual modern contraceptive practice at the time of the survey. It is also the most widely used andvaluable measure of the success of family planning programmes. Furthermore, it can beused to estimate the reduction in fertility attributable to contraception. In this study, ever use of contraceptive method was classified as (never use = 0, traditional/folk methods = 1, modern methods = 2).

### The independent variable of interest

#### Wealth quintile

Wealth index was used as a substitute for income due to the lack of credible information on incomes in the 2010, MDHS. This is in agreement with other studies using demographic and health surveys [4,8,9]. The wealth index is used in this study as a background characteristic. It was used as a proxy for measuring the long-term standard of living and its computation was based on data from the household’s ownership of consumer goods; dwelling characteristics; type of drinking water source; toilet facilities; and other characteristics that are related to a household’s socioeconomic status. To construct the index, each of these assets was assigned a weight (factor score) generated through principal component analysis, and the resulting asset scores were standardised in relation to a standard normal distribution with a mean of zero and standard deviation of one [15]. Each household was then assigned a score for each asset, and the scores were summed for each household. Individuals were ranked according to the total score of the household in which they resided [8]. The aggregate score was therefore disentangled into five categories as poorest, poorer, middle, richer and richest. Our study utilized the extreme wealth quintiles (poorest and richest).

#### Multivariate analysis

Multinomial logistic was used because the categories of the dependent variables are more than two (non users, used only traditional method and used modern method). The multinomial model uses maximum likelihood estimation to evaluate the probability of categorical membership of each type of contraceptive method used.

In view of the fact that the dependent variable has 3 categories, this requires the calculation of 3-1 = 2 equations, one for each category relative to the reference category (not using any contraceptive method), to describe the relationship between modern contraceptive use and the independent variables. We chose the first category (non users) as the reference, then, for n = 2,3

$$\ln\left\{\frac{p\left(\zeta_{i}=n\right)}{p\left(\zeta_{i}=1\right)}\right\}=\propto_{n}+\sum_{k=1}^{K}\beta_{\mathrm{nk}}X_{\mathrm{ik}}=Z_{\mathrm{ni}}$$

Hence, for each case, there will be 2 predicted log odds, one for each category relative to the reference category. When there are more than 2 groups, computing probabilities is a little more problematical than it was in logistic regression. For n = 2,3

$$p\left(\zeta_{i}=n\right)=\frac{\exp\left(Z_{\mathrm{ni}}\right)}{1+\sum_{\tau =2}^{3}\exp\left(Z_{\mathrm{\tau i}}\right)}$$

For the reference category;

$$p\left(\zeta_{i}=1\right)=\frac{1}{1+\sum_{\tau =2}^{3}\exp\left(Z_{\mathrm{\tau i}}\right)}$$

#### Estimation of Adjusted Total Fertility Rate

The widely used “Coale and Trussell P/F ratio model” indirect approach for estimating Total Fertility Rate (P/F ratio) was used to provide estimate of fertility for the women in the poorest and richest wealth quintiles. The approach was used as a result of inconsistencies and errors in the estimates produced through direct method [16]. The procedures in the estimation of conventional and adjusted total fertility rates are as shown below;

a) Average parities reported P(i): P(i) = Total number of children ever born to women in age group (i) divided by Total number of women in age group (i).

b) Preliminary fertility schedule f(i): f(i) = Number of births in the year preceding the survey in age group (i) divided by Total number of women in age group (i).

c) Cumulated fertility schedule for a period φ(i):

$$\varphi \left(i\right)=\sum_{k=0}^{i}f\left(k\right)$$

d) Average parity equivalents for a period (F(i)): F(i) are computed by interpolation using the period fertility rates f(i) and the cumulated fertility values φ(i).

(1) $$F\left(i\right)=\varphi \left(i-1\right)+a\left(i\right)f\left(i\right)+b\left(i\right)f\left(i+1\right)+c\left(i\right)\varphi \left(7\right)$$

e) Fertility Schedule for conventional five-year age groups **f**^+^(**i**) : **f**^+^(**i**) values are estimated by weighting the rates referring to unorthodox age groups using the equation below:

(2) $$f^{+}\left(i\right)=\left[1-w\left(i-1\right)\right]f\left(i\right)+w\left(i\right)f\left(i+1\right)$$

Where;

$$w\left(i\right)=x\left(i\right)+y\left(i\right)\times \frac{f\left(i\right)}{\varphi \left(7\right)}+z\left(i\right)\times \frac{f\left(i+1\right)}{\varphi \left(7\right)}$$

. The values of x(i), y(i) and z(i) are constants

f) Adjustment of period fertility schedule: This can be done by calculating the P/F ratios i.e. average parity divided by parity equivalent. Then, the adjusted age-specific fertility rates for conventional age groups f*(i) is estimated by simply multiplying the f^+^(i) values by the adjustment factor k.

g) The adjusted total fertility rate (TFR) is 5 × ∑ f*(i)

### Ethical considerations

Approval for data utilized for this study was obtained from the data originator, Micro International U.S.A before the data was extracted from their web platform. At the point of data collection by the data originators, an informed consent was sought from all the study participants after detailed description of all the issues related to the study were passed across to the respondents. Eligible respondents who did not want to participate in the study were excluded from the survey. Each consenting participants was made to sign appropriate agreement form before the commencement of the interview.

## Results

The mean children ever born of the women in the poorest (3.94±2.7) was higher than their counterparts in the richest (2.82±2.3) wealth quintile (p<0.001). It is alarming that none of the women in poorest wealth quintile had higher education. Table 1 shows that among all the women included in the analysis 75.5% ever used modern contraceptive, while 66.8% and 82.4% ever used modern contraceptive in the poorest and richest wealth quintile respectively (p<0.001). Among the religious group, 82.2% and 56.4% of women who belong to Catholic and Muslims ever used modern contraceptive. Ever use of modern contraceptive was significantly associated with; current age, education, age at first marriage, region, place of residence, children ever born and age at first birth (p<0.001).

**Table 1:** Distribution of ever use and current use of modern contraceptive according to background characteristics, Malawi, 2010

**Background**	**Ever used method**			**Current use**		
**Characteristics**	**Modern**	**Total**	***x***^**2**^**-value**	**Modern**	**Total**	***x***^**2**^**-value**
Total	75.6(3844)	5085		53.8(1232)	2289	
**Age**						
15-19	45.1(137)	304	295.270*	37.1(46)	124	32.10**
20-24	72.7(750)	1032		53.4(197)	369	
25-29	81.1(1062)	1310		53.4(282)	528	
30-34	82.6(749)	907		55.3(241)	436	
35-39	81.0(577)	712		59.5(238)	400	
40-44	71.1(320)	450		52.5(136)	259	
45-49	67.0(250)	373		53.8(93)	173	
**Education**						
No education	60.9(508)	834	179.95*	43.2(152)	352	57.56*
Primary	75.5(2218)	2939		53.3(672)	1261	
Secondary	84.8(980)	1156		60.8(355)	584	
Higher	88.0(139)	158		58.1(54)	93	
**Wealth quintile**						
Poorest	66.8(1503)	2251	177.95*	45.9(393)	856	46.68*
Richest	82.6(2341)	2834		58.5(839)	1433	
**Religion**						
Catholic	82.2(866)	1054	198.19*	55.8(288)	516	41.99*
Other Christians	77.6(2549)	3283		56.1(832)	1483	
Muslims	56.4(378)	670		36.6(93)	254	
Others	64.2(52)	81		56.8(21)	37	
**Age at 1**^**st **^**marriage**						
8-14	75.2(424)	564	31.76*	49.4(119)	241	17.50***
15-19	76.1(2481)	3260		55.9(801)	1433	
20-24	75.6(804)	1063		53.1(272)	512	
25-29	69.0(120)	174		40.0(36)	90	
30+	61.5(16)	26		42.9(6)	14	
**Total life time number of sexual partners**						
1	75.8(2209)	2913	1.838	59.2(753)	1273	39.57*
2+	75.3(1636)	2174		47.2(480)	1016	
**Age at first sexual intercourse**						
8-14	76.0(433)	570	11.419	55.0(144)	262	11.784
15-19	75.6(1766)	2337		52.0(549)	1056	
20-24	79.1(330)	417		57.7(113)	196	
25-33	77.8(28)	36		40.9(9)	22	
at first union	74.6(1287)	1726		55.4(416)	751	
**Region**						
Northern	72.1(393)	545	40.65*	56.5(140)	248	32.94*
Central	76.2(1647)	2162		54.5(542)	995	
Southern	75.8(1805)	2380		52.6(550)	1045	
**Place of residence**						
Urban	83.7(1291)	1542	80.74*	56.8(443)	780	6.98***
Rural	72.0(2554)	3545		52.3(790)	1510	
**Children ever born**						
None	27.4(69)	252	425.58*	7.5(8)	106	151.85*
1-2	73.9(1247)	1688		48.6(370)	762	
3-4	82.3(1251)	1520		58.0(374)	645	
5+	78.6(1278)	1626		61.9(481)	777	
**Age at first birth**						
10-14	74.7(186)	249	31.16*	50.0(48)	96	15.85***
15-19	80.0(2320)	2900		56.5(731)	1294	
20-24	76.3(1116)	1463		58.1(395)	680	
25-29	69.4(134)	193		45.5(45)	99	
30+	64.5(20)	31		46.2(6)	13	

*Significant at 0.1%; **Significant at 1%; ***Significant at 5%*.*

The prevalence of current use of modern contraceptive was 53.8%. The percentage of women who were currently using contraceptive was significantly higher among richest (58.5%) than the poorest (45.9%). Differential in current use of modern contraceptive also existed between religious group (p<0.001), age at first marriage (p=0.0250), total life time number of sexual partner (p<0.001), regions (p<0.001), place of residence (p=0.031). As expected, current use of modern contraceptive was more prominent among married women who have given birth to at least 5 children (61.9%) than those who had no children (7.5%) and those who already gave birth to 1–2 children (48.6%) (p<0.001). Also significant variation existed in current use of modern contraceptive among subgroup of women with respect to age at first birth (p=0.045), current age (p=0.001) and level of education (p<0.001).

Using multiple logistic regressions as shown in Table 2, the data reveals that women in richest wealth quintile were more likely (OR=2.36, C.I=2.07-2.69) to ever use modern contraceptive than their counterparts in the poorest wealth quintile (model 1a: using wealth index as the sole independent variable). The odds of ever use of modern contraceptive of those in richest wealth quintile reduces (OR=1.85, C.I=1.55-2.22) when religion, levels of education, region and place of residence were used as control (model 2a). However, slight reduction existed in the odd ratio of women in richest wealth quintile (OR=1.85, C.I=1.51-2.26) when other variables such as current age, age at first birth, age at first marriage, and children ever born were added to the regression model (model 3a).

**Table 2:** Multinomial logistic regression of relationship between ever use of modern contraceptive and background characteristics, Malawi, 2010

**Background characteristics**	**Model 1a**		**Model 2a**		**Model 3a**	
	**UOR**	**C.I (UOR)**	**AOR**	**C.I (UOR)**	**AOR**	**C.I (UOR)**
		**Lower-upper**		**Lower-upper**		**Lower-upper**
**Wealth index**						
Poorest	1		1		1	
Richest	2.36*	2.07-2.69	1.85*	1.55-2.22	1.85*	1.51-2.26
**Education**						
None			1		1	
Primary			1.55*	1.30-1.84	1.72*	1.42-2.09
Secondary			1.93*	1.50-2.47	2.80*	2.08-3.77
Higher			2.04*	1.21-3.44	5.43*	2.68-10.97
**Residence**						
Urban			1		1	
Rural			0.90	0.74-1.09	0.82	0.66-1.02
**Religion**						
Catholic			1		1	
Other Christians			0.79***	0.66-0.95	0.84	0.69-1.02
Muslims			0.30*	0.24-0.38	0.31*	0.24-0.40
Others			0.57***	0.35-0.94	0.52***	0.30-0.88
**Region**						
Northern			1		1	
Central			1.70*	1.36-2.14	1.68*	1.30-2.16
Southern			1.76*	1.40-2.22	1.69*	1.31-2.18
**Age at first marriage**						
8-14					1	
15-19					0.95	0.73-1.23
20-24					0.85	0.60-1.19
25-29					0.58	0.33-1.02
30+					0.80	0.28-2.28
**Age at first birth**						
10-14					1	
15-19					1.08	0.75-1.57
20-24					0.66***	0.44-0.99
25-29					0.57***	0.32-0.99
30+					0.69	0.25-1.92
**Children ever born**						
None					1	
1-2					1.84*	1.48-2.30
3-4					2.82*	2.10-3.78
5+					3.17***	2.34-4.37
**Age as at last birthday**						
15-19					1	
20-24					2.44*	1.75-3.40
25-29					3.33*	2.37-4.66
30-34					3.37*	2.32-4.90
35-39					3.02*	2.01-4.55
40-44					1.62***	1.06-2.49
45-49					1.31	0.84-2.05
*−2 Loglikelihood*	*5484.24*		*5288.61*		*4547.01*	
*Nagelkerke R*^*2*^	*0.049*		*0.103*		*0.162*	

*Significant at 0.1%; **Significant at 1%; ***Significant at 5%; *C.I: Confidence interval for exp(β)*; *UOR: Unadjusted odd ratio*, *AOR: adjusted odd ratio*.

The data further show that the odd ratio of ever use of modern contraceptive use increases with increasing level of education. For instance, women who had higher education were 5.4 times more likely to ever use modern contraceptive than their counterparts with no formal education (p<0.001). Women that belong to Muslim religious group were 0.313(C.I=0.24-0.40) times less likely to ever use modern contraceptive than Catholic women. Ever use of modern contraceptive was more prominent among women who reside in central (OR=1.68, C.I=1.30-2.16) and southern regions (OR=1.69, C.I=1.31-2.18) than northern religion. The data is also evidenced that women who had their first birth at ages 20–24 (OR=0.66, C.I=0.44-0.99) and 25–29 (OR=0.57, C.I=0.32-0.99) years were less likely to ever use modern contraceptive than those had their first birth at age 10–14 years. The odd ratio of ever use of modern contraceptive increases with number of children previously born. As an example, women who already gave birth to at least 5 children were 3.167(p=0.025) more likely to ever use modern contraceptive than those who had never had a child. See Table 2 for more details of the relationship between socio-demographic factors and modern contraceptive use.

As shown in Table 3, restricting the analysis to wealth quintile and current use of modern contraceptive, the logistic regression revealed that women in richest wealth quintile were 1.66(C.I=1.40-1.97) more likely to currently use modern contraceptive than those in poorest (model1b). The odds of currently using modern contraceptive among richest wealth quintile slightly varies (OR=1.61, C.I=1.28-2.02) when religion, levels of education, region and place of residence were used as control (model 2b). The odd ratio of current use of modern contraceptive among the women in richest wealth quintile (OR=1.54, CI=1.12-1.97) reduced considerably when other variables such as age, age at first birth, age at first marriage, and children ever born were added to the regression model (model 3b). However, in this model, women in richest wealth quintile were currently using modern contraceptive at higher ratio than their counterparts in poorest wealth quintile. Also, the odd ratio of currently using modern contraceptive falls consistently with increasing age group, but reduces as the number of children ever born increases. Also, women with more than one life-time number of sexual partners were less likely 0.636 (p<0.0001) to currently using modern contraceptive than those with only one life-time number of sexual partner.

**Table 3:** Multinomial logistic regression of relationship between currently using modern contraceptive and background characteristics, Malawi, 2010

**Background characteristics**	**Model 1b**		**Model 2b**		**Model 3b**	
	**UOR**	**C.I (UOR)**	**AOR**	**C.I (UOR)**	**AOR**	**C.I (UOR)**
		**Lower-upper**		**Lower-upper**		**Lower-upper**
**Wealth index**						
Poorest	1		1		1	
Richest	1.66*	1.40-1.97	1.61*	1.28-2.02	1.54**	1.12-1.97
**Education**						
None			1		1	
Primary			1.24	0.96-1.59	1.26	0.96-1.66
Secondary			1.42***	1.04-1.94	2.15*	1.50-3.10
Higher			1.24	0.75-2.04	2.91**	1.59-5.31
**Residence**						
Urban			1		1	
Rural			1.16	0.94-1.45	0.97	0.77-1.23
**Religion**						
Catholic			1		1	
Other christians			1.06	0.87-1.30	1.06	0.85-1.31
Muslims			0.53*	0.38-0.73	0.59**	0.42-0.82
Others			1.31	0.66-2.61	1.35	0.65-2.83
**Region**						
Northern			1		1	
Central			1.12	0.84-1.50	1.17	0.85-1.60
Southern			1.05	0.78-1.41	1.20	0.87-1.66
**Age at first marriage**						
8-14					1	
15-19					1.16	0.82-1.65
20-24					0.87	0.56-1.34
25-29					0.82	0.39-1.71
30+					0.94	0.25-3.46
**Age at first birth**						
10-14						
15-19					1.12	0.66-1.87
20-24					1.36	0.77-2.39
25-29					1.22	0.57-2.63
30+					1.40	0.34-5.75
**Children ever born**						
None					1	
1-2					2.17*	1.23-3.01
3-4					2.42*	1.84-3.19
5+					4.72*	3.30-6.73
**Age at last birthday**						
15-19					1	
20-24					0.77	0.46-1.28
25-29					0.46**	0.27-0.78
30-34					0.34*	0.19-0.60
35-39					0.32*	0.18-0.59
40-44					0.23*	0.12-0.44
45-49					0.22*	0.11-0.44
**Total life time number of sexual partner**						
1					1	
2+					0.64*	0.529-0.76
*−2 Loglikelihood*	*3125.73*		*3088.85*		*2800.57*	
*Nagelkerke R2*	*0.020*		*0.041*		*0.114*	

*Significant at 0.1%; **Significant at 1%; ***Significant at 5%; *C.I: Confidence interval for exp(β)*; *UOR: Unadjusted odd ratio*, *AOR: adjusted odd ratio*.

**Figure 2:**
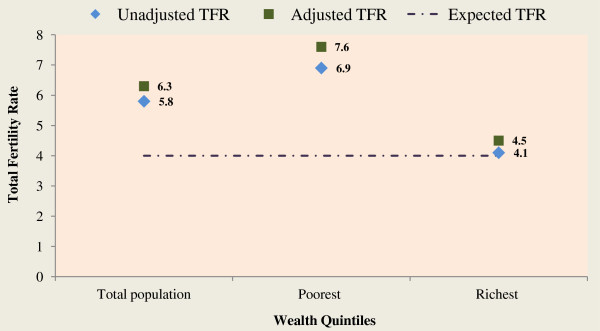
Unadjusted and adjusted total fertility rates for total population, poorest and richest wealth quintiles, Malawi, 2010.

**Figure 3:**
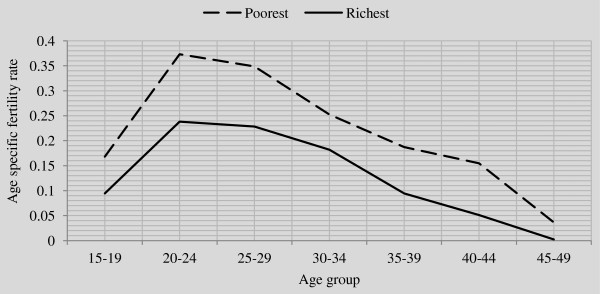
Line graph of adjusted age specific fertility rates for women in poorest and richest wealth quintile in Malawi.

The data as shown in Tables 4, 5 and 6 revealed that the reported period total fertility rate (TFR) was strikingly higher among women in poorest (TFR=6.85) wealth quintile than the richest (TFR=4.12) and national estimate (TFR=5.76). Consistently, the adjusted total fertility rate of women in all the three segments of the population (total population, women in poorest and richest wealth quintiles) considered in this study were higher than the conventional estimates (unadjusted).In Figure 2, the expected total fertility rate (TFR=4) line was drawn to see the gap between the attained TFR (either adjusted or not) for women in the poorest and richest wealth quintiles.Figure 3 is an indication that across all the five year age groups, fertility rate was higher among women in the poorest wealth quintile than their counterparts in the richest wealth quintile.

**Table 4:** Reported period and adjusted total fertility rates for conventional age groups, total population, Malawi, MDHS, 2010

**Age**	**FP(i)**	**CEB(i)**	**BIPY**	**P(i)**	**f(i)**	**φ(i)**	**F(i)**	**f**^**+**^**(i)**	**P/F**	f*(i)
**Total population**										
15-19	5005	1140	556	0.2278	0.1111	0.5555	0.2400	0.1347	0.9490	0.1480
20-24	4554	7349	1332	1.6137	0.2925	2.0180	1.4329	0.2958	1.1262	0.3249
25-29	4400	13095	1090	2.9761	0.2477	3.2565	2.7815	0.2437	1.0700	0.2678
30-34	3250	13750	668	4.2308	0.2055	4.2840	3.8902	0.2016	1.0875	0.2214
35-39	2521	13734	405	5.4478	0.1607	5.0875	4.7759	0.1566	1.1407	0.1720
40-44	1730	10830	174	6.2601	0.1006	5.5905	5.3894	0.0927	1.1616	0.1018
45-49	1558	10774	54	6.9153	0.0347	5.7640	5.7236	0.0277	1.2082	0.0304
Total	23018				1.1527					1.2664
Total fertility rate	**5.7635**					**6.332**				

*FP(i)*: *Women’s population;CEB(i): Children ever born;BIPY: Births in the past one year prior the survey;P(I): Average parities reported;f(i): Preliminary fertility schedule;φ(i): Cumulated fertility schedule for a period;F(i): Average parity equivalents for a period;****f***^*+*^***(i)***: Fertility Schedule for conventional five-year age groups;f*(i): The adjusted total fertility rate.

**Table 5:** Reported period and adjusted total fertility rates for conventional age groups, total population, women in POOREST wealth quintiles, Malawi, MDHS, 2010

**Age**	**FP(i)**	**CEB(i)**	**BIPY**	**P(i)**	**f(i)**	**φ(i)**	**F(i)**	**f**^**+**^**(i)**	**P/F**	f*(i)
**Poorest wealth quintile**										
15-19	892	247	112	0.2769	0.1256	0.6280	0.2726	0.1513	1.0160	0.1680
20-24	820	1554	269	1.8951	0.3280	2.2680	1.5862	0.3359	1.1948	0.3730
25-29	793	2650	254	3.3417	0.3203	3.8695	3.2662	0.3142	1.0231	0.3489
30-34	602	2837	141	4.7126	0.2342	5.0405	4.5998	0.2272	1.0245	0.2523
35-39	482	2792	82	5.7925	0.1701	5.8910	5.5291	0.1685	1.0476	0.1871
40-44	348	2357	52	6.7730	0.1494	6.6380	6.3634	0.1394	1.0644	0.1548
45-49	334	2406	14	7.2036	0.0419	6.8475	6.7995	0.0330	1.0594	0.0366
Total	4271				1.3696					1.5208
Total Fertility Rate	**6.848**					**7.604**				

*FP(i)*: *Women’s population;CEB(i): Children ever born;BIPY: Births in the past one year prior the survey;P(I): Average parities reported;f(i): Preliminary fertility schedule;φ(i): Cumulated fertility schedule for a period;F(i): Average parity equivalents for a period;f*^***+***^***(i)***: Fertility Schedule for conventional five-year age groups;f*(i): The adjusted total fertility rate.

**Table 6:** Reported period and adjusted total fertility rates for conventional age groups, total population, women in the richest wealth quintiles, Malawi, MDHS, 2010

**Age**	**FP(i)**	**CEB(i)**	**BIPY**	**P(i)**	**f(i)**	**φ(i)**	**F(i)**	**f**^**+**^**(i)**	**P/F**	f*(i)
**Richest wealth quintile**										
15-19	1254	167	89	0.1332	0.0710	0.3550	0.1494	0.0873	0.8914	0.0943
20-24	1073	1110	229	1.0345	0.2134	1.4220	0.9687	0.2202	1.0679	0.2380
25-29	1098	2485	235	2.2632	0.2140	2.4920	2.0699	0.2114	1.0934	0.2284
30-34	754	2516	133	3.3369	0.1764	3.3740	3.0596	0.1683	1.0906	0.1819
35-39	558	2504	51	4.4875	0.0914	3.8310	3.6564	0.0874	1.2273	0.0945
40-44	348	1769	19	5.0833	0.0546	4.1040	4.0333	0.0472	1.2604	0.0510
45-49	303	1819	1	6.0033	0.0033	4.1205	4.1167	0.0023	1.4583	0.0025
Total	5388				0.8241					0.8907
Total fertility rate	**4.1205**					**4.4535**				

*FP(i)*: *Women’s population;CEB(i): Children ever born;BIPY: Births in the past one year prior the survey;P(I): Average parities reported;f(i): Preliminary fertility schedule;φ(i): Cumulated fertility schedule for a period;F(i): Average parity equivalents for a period;****f***^***+***^(i): Fertility Schedule for conventional five-year age groups;f*(i): The adjusted total fertility rate.

## Discussion

The use of modern contraceptives is known to be highly cost-effective and has demonstrable poverty-reducing effects in earlier studies [17-21]. Modern contraceptives also help women achieve their human rights to health, autonomy, and personal about family size [4]. Despite its tremendous benefits and efforts of government and agencies, funding from donors and policymakers did not keep pace with the growing need. In many countries around the world today, high fertility and rapid population growth continue to jeopardize their socioeconomic advancement.

Most studies have found a strong association between wealth quintiles and modern contraceptive use. However, among these studies, very few have restricted their focus on the two extreme classes of wealth quintile as evidenced in our study [22-25]. Our finding corroborates the significance of salient differential in wealth quintile in explaining the utilization of modern contraceptives and fertility in Malawi.

The study reveals that the mean children ever born and adjusted Total Fertility Rate (TFR) were higher among women in the poorest than those in the richest wealth quintile. Also, across all the 5-year age groups, fertility rate was consistently higher among women in poorest wealth quintile than those in the richest wealth quintile. This is consistent with findings from previous studies in Malawi and other parts of the world [22,26-29]. However, our finding may be a result of wealth inequality among women in Malawi. Inequality in peoples’ wealth can influence their socioeconomic status including access to modern health care and education [26,30]. It is also evidenced in the current study that none of the women in the poorest wealth quintile has higher education. This finding may have a lot of socioeconomic and health implications on the life of women in the poorest wealth quintile in Malawi. The adjusted TFR estimate of women in richest wealth quintile (4.45) is an indication of change in fertility towards attainment of childbearing limit of 4 per woman in Malawi whereas, the poorest women’s TFR (7.60) is still quite far from reaching the goal.

This study further revealed that higher proportion of women in the richest wealth quintile ever used and currently using modern contraceptive method than their counterparts in the poorest. Modern family planning services at times may involve some financial obligations on the part of the users, particularly when such services are not free or the service providers are at far locations from the residence of women who intend to utilize them. For instance, a study conducted by Bongaarts et al. and Tuoane et al. show that free access to family planning services predisposed people to use of modern contraceptive [4,31]. The result from our study is in line with the findings from studies previously conducted in Nigeria, sub-Saharan Africa [7,32].

Our study further shows that among the religious group higher proportion of women who are Christians either ever used or currently using modern contraceptive than those belonging to Islamic religion. Different studies across the globe have upheld the same finding [7,23,33,34].

Our finding also gives credence to the differential in levels of education in the utilization of modern contraceptives. The prevalence of ever and current use of modern contraceptive increased with increasing level of education. The influence of education on modern contraceptive use cannot be over-emphasized. This is because, as the level of education increases, wealth and prestige tend to increase and the intention to limit children by using modern contraceptive will increase. Apparently, education leads to a greater ability to acquire wealth and prestige; this competes with decision on childbearing since in a modern economy children are for many years resource consumers rather than resource producers. This principle seems to work for individuals as they attempt to be upwardly mobile socially within a society, or as they try to prevent social slippage; a loss of social and economic status relative to others.

Conscious of the implications of high fertility on the health and socioeconomic status of women, in this study, in addition we explored the association between modern contraceptive use and children ever born. For instance, when fertility levels are high, women’s lives are subjugated by a repeated series of pregnancy, breastfeeding, and nurture of young children [35]. It is interesting to know that the proportion of women who had ever used or currently using modern contraceptive increases with increasing number of children ever born. This is an indication that realization of women to halt childbearing is in progress in Malawi.

The result of the influence of age on modern contraceptive use which shows lower use among adolescent women included in this study than other age segments is expected. This could be attributed to the fact that majority of adolescent women began sexual initiation not quite long and are yet to acquaint themselves with contraception and sexual health [36]. Besides, in African context, young women are forbidden to talk about sexual issues in the society and as such this could hinder their access to family planning practices. Our finding is in conformity with previous studies among women in United States of America and Canada [37,38].

In anticipation to link the relationship between wealth quintile and modern contraceptive use, we generated three models with the use of wealth quintile as the sole independent variable; wealth quintile with religion, levels of education, region and place of residence as control; wealth quintile with further variables as control. Across the three models, the data revealed strong effect of richest wealth quintile on either ever use or current use of modern contraceptive in Malawi. Moreover, the identified predictors of ever use of modern contraceptive are; wealth quintile, education, religion, children ever born, age at first birth and current age. For current use, the predictors are; wealth quintile, education, religion, children ever born, current age and total life-time number of sexual partners. The observed findings from the multinomial logistic regression are consistent with what is obtainable in the literature [22].

### Limitations

The data is secondary and as such the shortcomings of outcome of such data cannot be completely ruled out from our study. Also, information on events that occur in the past may be susceptible to errors as a result of recall bias or memory lapses. For instance, in this study, information were sought from women on whether they ever use modern contraceptive or not, older women may not remember the event particularly, if they had used modern contraceptive a long time ago. Despite these limitations, the data originator put appropriate mechanisms in place to ensure accurate and reliable data at the point of collection.

## Conclusion

Wide differential exists in fertility and modern contraceptive use between women in the richest and poorest wealth quintile in Malawi. Women in richest wealth quintile have almost attained the goal of limiting births to at most 4 children per woman while achieving this target is still a mirage for women in the poorest wealth quintile. The need for investing in education of the poor is urgent. This will have positive effect on modern contraceptive use and fertility reduction among the poorest women. The poorest individuals and those with unmet need for family planning should be reached on a wide scale. Government should reduce inequities in access and use, such as those related to wealth quintile, education, religion, children ever born, age at first birth and age. Further research is required to examine reasons for non-use of modern contraceptive method across all the wealth quintiles in Malawi.

## Competing interests

The authors declare that they have no competing interests.

## Authors’ contributions

SAA (Adebowale) conceived the idea, wrote the study background and methodology section, did the data extraction, data analysis and interpretation. SAA (Adedini) and LI reviewed the data analysis and wrote the discussion. MEP reviewed relevant literatures and the manuscript. All the authors reviewed and approved the final draft of the manuscript.

## Pre-publication history

The pre-publication history for this paper can be accessed here:

http://www.biomedcentral.com/1472-6874/14/40/prepub
